# Acromegaloid Facial Appearance: Case Report and Literature Review

**DOI:** 10.1155/2013/970396

**Published:** 2013-02-28

**Authors:** Adline Ghazi, Shikha Khosla, Kenneth Becker

**Affiliations:** ^1^Diabetes Care Program, Medstar Good Samaritan Hospital, 5601 Loch Raven Blvd, Baltimore, MD 21239, USA; ^2^Division of Endocrinology, Washington DC Veterans Affairs Medical Center (DCVAMC), George Washington University, Washington, DC, USA

## Abstract

Pseudoacromegaly is characterized by an acromegalic appearance without any abnormality of growth hormone function. It may be caused by several congenital and acquired conditions. One such condition is the acromegaloid facial appearance (AFA) syndrome. This condition has been described in approximately eight cases/families. It encompasses a spectrum of acromegaloid physical findings, normal growth hormone (GH) and insulin-like growth factor one (IGF-1) levels, and variable mode of inheritance. The most common physical findings are coarse facies, bulbous nose, and thickened lips. We present a case and a review of the literature on this illness. The patient is a 57-year-old woman who was referred to the endocrinology division for evaluation of suspected acromegaly. She had an acromegaloid appearance since birth as well as a terminal hypertrichosis. Her endocrine laboratory evaluation and chromosomal analyses were normal. AFA needs to be considered when evaluating any patient with pseudoacromegaly. Additional cases/families need to be identified in order to better understand the clinical spectrum, clinical implications, and mode of inheritance of AFA.

## 1. Introduction

Acromegaly is characterized by skin and soft tissue changes due to increased growth hormone levels. Patients with similar physical findings but with an intact somatotroph axis are considered to have pseudoacromegaly. Another term used in the literature to describe this condition is acromegaloidism [1]. These latter patients have normal serum IGF-1 levels and reveal a suppressed serum GH following an oral glucose challenge. Conditions responsible for pseudoacromegaly include, among others, severe insulin resistance, pachydermoperiostitis, Ascher's syndrome, multiple neuromas syndrome, drug intake (e.g., Minoxidil, Phenytoin), and hypothyroidism [2, 3]. Pseudoacromegaly due to severe insulin resistance is due to supraphysiologic levels of insulin that stimulate growth through an intact mitogenic signaling pathway. The underlying etiology for excess soft tissue growth in other conditions is not known but is probably due to growth factors different from those of GH and IGF-1 [4]. Another cause of pseudoacromegaly is the syndrome referred to as acromegaloid facial appearance (AFA) and a variance of it, which includes terminal hypertrichosis. This syndrome has been reported so far in about eight cases/families that demonstrate an acromegaloid appearance [5–12]. Interestingly, patients with AFA have differing inheritance. The majority of cases seem to be inherited in an autosomal dominant pattern or autosomal dominant pattern with incomplete penetrance.

## 2. Case Report

A 57-year-old woman was referred to the endocrine service because of suspected acromegaly. She had been also seen for the same concern approximately 20 years previously and had been told that “everything was normal.” She complained of chronic headaches for 25 years and has tried several medications with partial relief. She reported having a “peculiar appearance” since infancy. She always had a deep voice and was very hirsute. She has been plucking her facial hair (upper lip and chin) for many years. She also complained of thick black hair on her arms and legs, and some in her lower back and lower abdomen. She had two miscarriages: one tubal pregnancy and one successful pregnancy 35 years prior. This son was born premature and had a patent ductus arteriosus. She denied any change in the size of her shoes, gloves, or rings. She had no history of learning disability.

Her past medical history included severe osteoarthritis, chronic headaches, Barrett's esophagus, osteopenia, and insomnia. Her menopause commenced at age 44.

As for her family, she was known to resemble her son (Figure 3), paternal grandfather, and her father's paternal aunt. Her ancestry is Irish, German, and Native American Indian.

**Figure 1:**
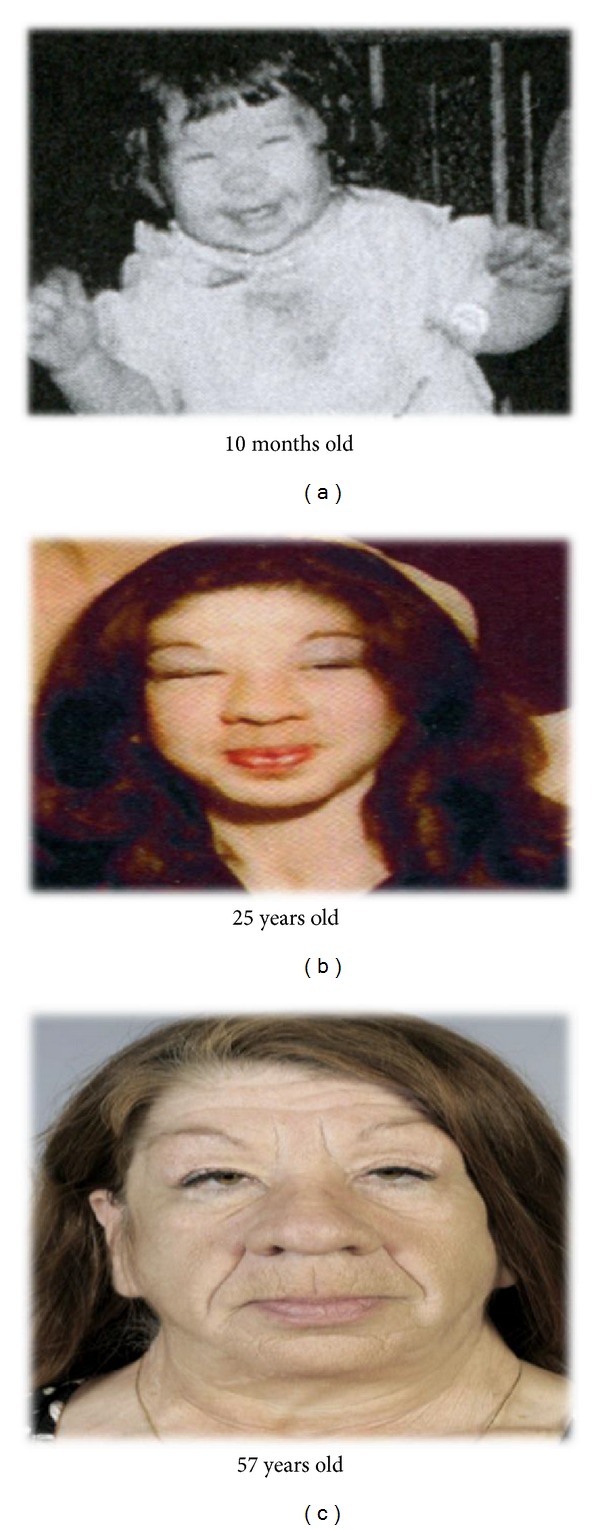
Coarse facies and bulbous nose, present since childhood.

**Figure 2:**
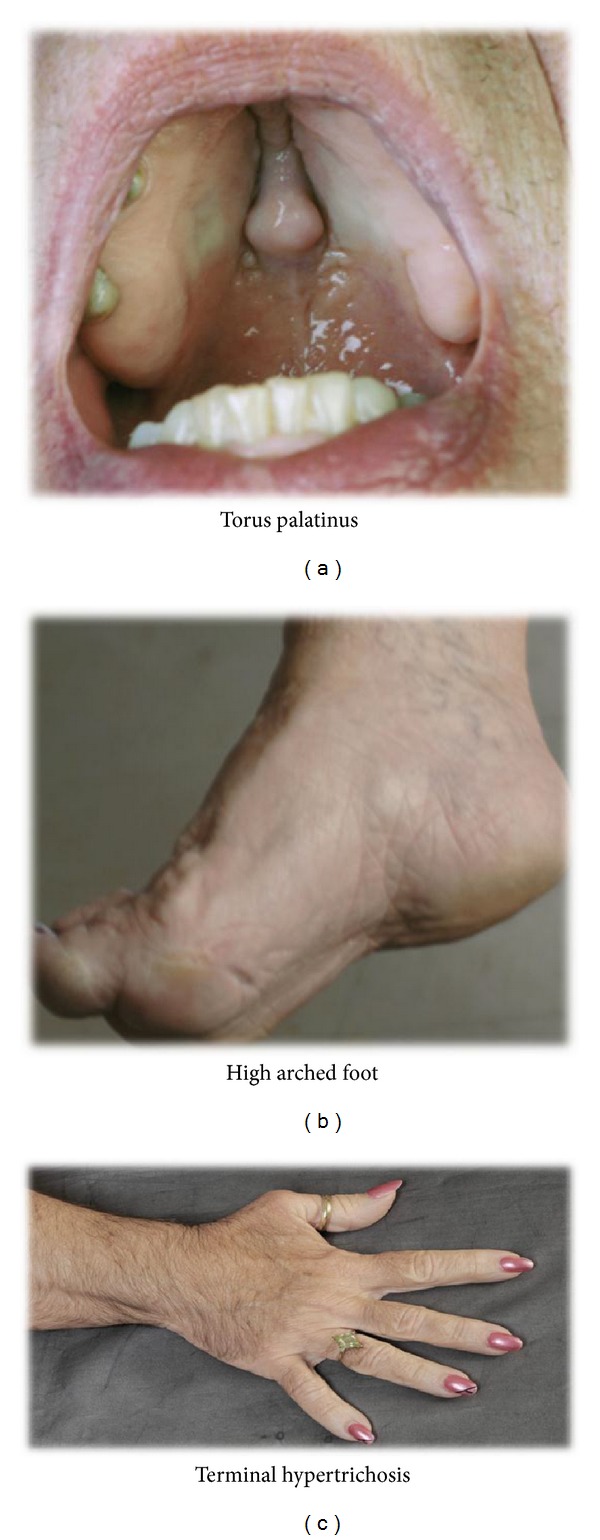
Other physical features.

**Figure 3:**
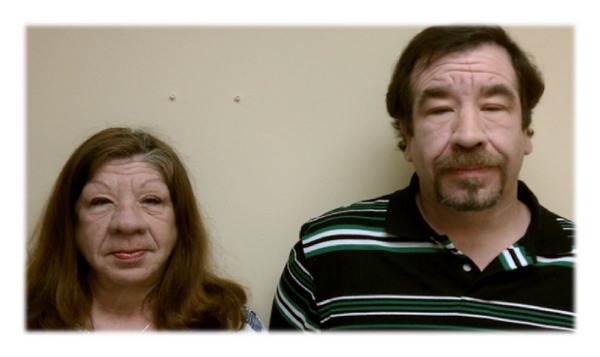
Patient and her son (37 years old).

Examination revealed an intelligent woman of stated age, short stature with a broad bulbous nose, and coarse facies with thick facial skin. She had deep nasolabial folds, marked furrows of the brow, blepharophimosis, drooping of eyelids, high arched eyebrows, thickened lower lip, and a torus palatinus. She has increased hair growth on upper lip, chin, and coarse terminal hair of arms and legs (Ferriman-Gallwey Score > 12) (Figures 1 and 2). She had a Tanner 5 development without virilization. There was no macrocephaly, macroglossia, or clubbing of the fingers.

Laboratory data showed a testosterone level of 11 ng/dL and insulin-like growth factor-1 level (IGF-1) of 98 ng/mL. Baseline growth hormone was 0.4. Following a 75-gram oral glucose challenge, GH levels were suppressed to 0.1 at 60 minutes and 0.2 at 120 minutes. The glycosylated hemoglobin was 5.5% and TSH was 1.65 mcIU/mL (Table 1). High-resolution chromosomal analysis revealed no abnormalities.

## 3. Review of the Literature

Acromegaloid facial appearance (AFA) is one of the causes of pseudoacromegaly. AFA was first described in 1985 by Hughes et al. as a condition with thick lips, prominent rugae and frenula inside the mouth, blepharophimosis, high arched eyebrows, bulbous nose, and large hands and feet. They reported a large kindred of 13 affected family members spanning at least five generations. The phenotypes in the family members were variable. The pattern of inheritance suggested autosomal dominant inheritance [5]. After a further literature review, we found 8 other cases/families described with AFA or its variant, AFA with accompanying terminal hypertrichosis appearance [5–12]. These cases and/or families have a variable spectrum of physical findings, variable mode of inheritance, and normal GH and IGF-1 levels (Table 2). The mode of inheritance in most of these families with AFA seemed to be either autosomal dominant or autosomal dominant with incomplete penetrance. Zen et al. suggested that the mode of inheritance of their reported family was autosomal recessive based on the consanguinity of the parents [11]. Hughes et al. attempted to map the gene responsible for the AFA syndrome but were not successful [5]. However, Stratakis et al. identified pericentric inversion of chromosome 11 in their proband as well as 2 family members that had similar phenotypic features. This chromosomal anomaly was absent in the other family members who did not share these physical features [9]. Kini and Clayton-Smith report normal chromosomal analysis [12].

## 4. Discussion

Our case resembles some previously reported cases on AFA and hypertrichosis. This case is the fourth case described having this additional finding of hypertrichosis, which seems to be a variant of the AFA syndrome. The mode of inheritance in this case appears to be autosomal dominant with incomplete penetrance. AFA syndrome, with or without hypertrichosis, needs to be considered in the differential diagnosis of pseudoacromegaly. More cases/families need to be studied in order to better understand the pathophysiology and clarify the clinical manifestations, mode of inheritance, and chromosomal karyotypes. The etiology of excess soft tissue growth in these cases is not due to excess growth hormone or IGF-1 and could possibly be from another peptide that promotes growth. Interestingly, Ashcraft et al. analyzed sera from five patients with acromegaloidism and found a substance with an approximate molecular weight of 70000 dalton. This substance had growth-promoting activity in an erythroid colony formation assay (using cells from both normals and Laron dwarfs). It was shown to be independent of epidermal, nerve, or fibroblast growth factors and growth hormone [13]. This, to our knowledge, has not yet been replicated.

## 5. Conclusion

We present a case of pseudoacromegaly secondary to AFA syndrome with the additional manifestation of terminal hypertrichosis. This case adds to what is already known about this syndrome that constitutes an interesting subtype of pseudoacromegaly. More cases need to be identified and worked up to have a better understanding of the etiology, genetics, and clinical implications of this condition.

## Figures and Tables

**Table 1:** Laboratory tests.

Test	Result	Normal range
Testosterone	11 ng/dL	18–69
Insulin-like growth factor-1 level (IGF-1)	98 ng/mL	92–190
Growth hormone (GH) suppression test after a 75-gram oral glucose challenge	Baseline: 0.4 ng/mL After 60 mins: 0.1 ng/mL After 120 mins: 0.2 ng/mL	<10
High-resolution chromosomal analysis	No chromosomal abnormalities	
TSH	1.65 mcIU/mL	0.1–5

**Table 2:** A review of the reported cases/families with AFA.

	Present case	Hughes et al. [5]	Dallapiccola et al. [6]	Irvine et al. [7]	Da-Silva et al. [8]	Stratakis et al. [9]	Zelante et al. [10]	Zen et al. [11]	Kini and Clayton-Smith [12]
Coarse facies	+	+	+	+	+	+	+	+	+
Bulbous nose	+	+	+	+	+	+	+	+	+
Thickened lips	+	+	+	+	+	+	+	+	+
Narrow palpebral fissure	+	+	+	−	+	+	+	+	+
Thick intraoral mucosa	−	+	+	−	+	−	+	−	+
Large hands	−	+	+	−	−	−	+	+	−
Hyperextensible joints	−	+	+	−	−	−	−	−	+
High arched eyebrows	+	+	−	−	−	−	−	−	−
Recurrent pericardial effusions	−	−	−	Father of proband	−	−	+	−	−
Low IQ/learning disabilities	−	−	+	UNK	UNK	UNK	−	UNK	+
Terminal hypertrichosis	+	−	−	+	−	−	+	+	−
Mode of inheritance	IP	AD	AD	AD	UNK	IP	UNK	?AR	IP
Chromosomal anomalies	None	Not done	Not done	Not done	Not done	+	Not done	Not done	None

−: absent, +: present, UNK: unknown, AD: autosomal dominant, AR: autosomal recessive and IP: incomplete penetrance.

## References

[1] Mims RB (1978). Pituitary function and growth hormone dynamics in acromegaloidism. *Journal of the National Medical Association*.

[2] Hari Kumar KVS, Shaikh A, Anwar I, Prusty P (2012). Primary hypothyroidism presenting as pseudoacromegaly. *Pituitary*.

[3] Nguyen KH, Marks JG (2003). Pseudoacromegaly induced by the long-term use of minoxidil. *Journal of the American Academy of Dermatology*.

[4] Flier JS, Moller DE, Moses AC (1993). Insulin-mediated pseudoacromegaly: clinical and biochemical characterization of a syndrome of selective insulin resistance. *Journal of Clinical Endocrinology and Metabolism*.

[5] Hughes HE, McAlpine PJ, Cox DW, Philipps S (1985). An autosomal dominant syndrome with “acromegaloid” features and thickened oral mucosa. *Journal of Medical Genetics*.

[6] Dallapiccola B, Zelante L, Accadia L, Mingarelli R (1992). Acromegaloid facial appearance (AFA) syndrome: report of a second family. *Journal of Medical Genetics*.

[7] Irvine AD, Dolan OM, Hadden DR, Stewart FJ, Bingham EA, Nevin NC (1996). An autosomal dominant syndrome of acromegaloid facial appearance and generalised hypertrichosis terminalis. *Journal of Medical Genetics*.

[8] da-Silva EO, Duarte AAR, Andrade EJL, Furtado GJ (1998). A new case of the acromegaloid facial appearance syndrome?. *Clinical Dysmorphology*.

[9] Stratakis CA, Turner ML, Lafferty A (2001). A syndrome of overgrowth and acromegaloidism with normal growth hormone secretion is associated with chromosome 11 pericentric inversion. *Journal of Medical Genetics*.

[10] Zelante L, Gasparini P, Savoia A, Lomuto M, Pellicano R (2000). A new case of Acromegaloid Facial Appearance (AFA) syndrome with an expanded phenotype. *Clinical Dysmorphology*.

[11] Zen PRG, Schwartz IVD, Paskulin GA (2004). Acromegaloid facial appearance and hypertrichosis: a case suggesting autosomal recessive inheritance. *Clinical Dysmorphology*.

[12] Kini U, Clayton-Smith J (2004). Acromegaloid facial appearance syndrome: a further case report. *Clinical Dysmorphology*.

[13] Ashcraft MW, Hartzband PI, Van Herle AJ (1983). A unique growth factor in patients with acromegaloidism. *Journal of Clinical Endocrinology and Metabolism*.

